# Supplementary figures and images for: Human population and socioeconomic modulators of conservation performance in 788 Amazonian and Atlantic Forest reserves
Source: PeerJ. 2016 Jul 14;4:e2206. doi: 10.7717/peerj.2206 (PMC4950577; doi:10.7717/peerj.2206)

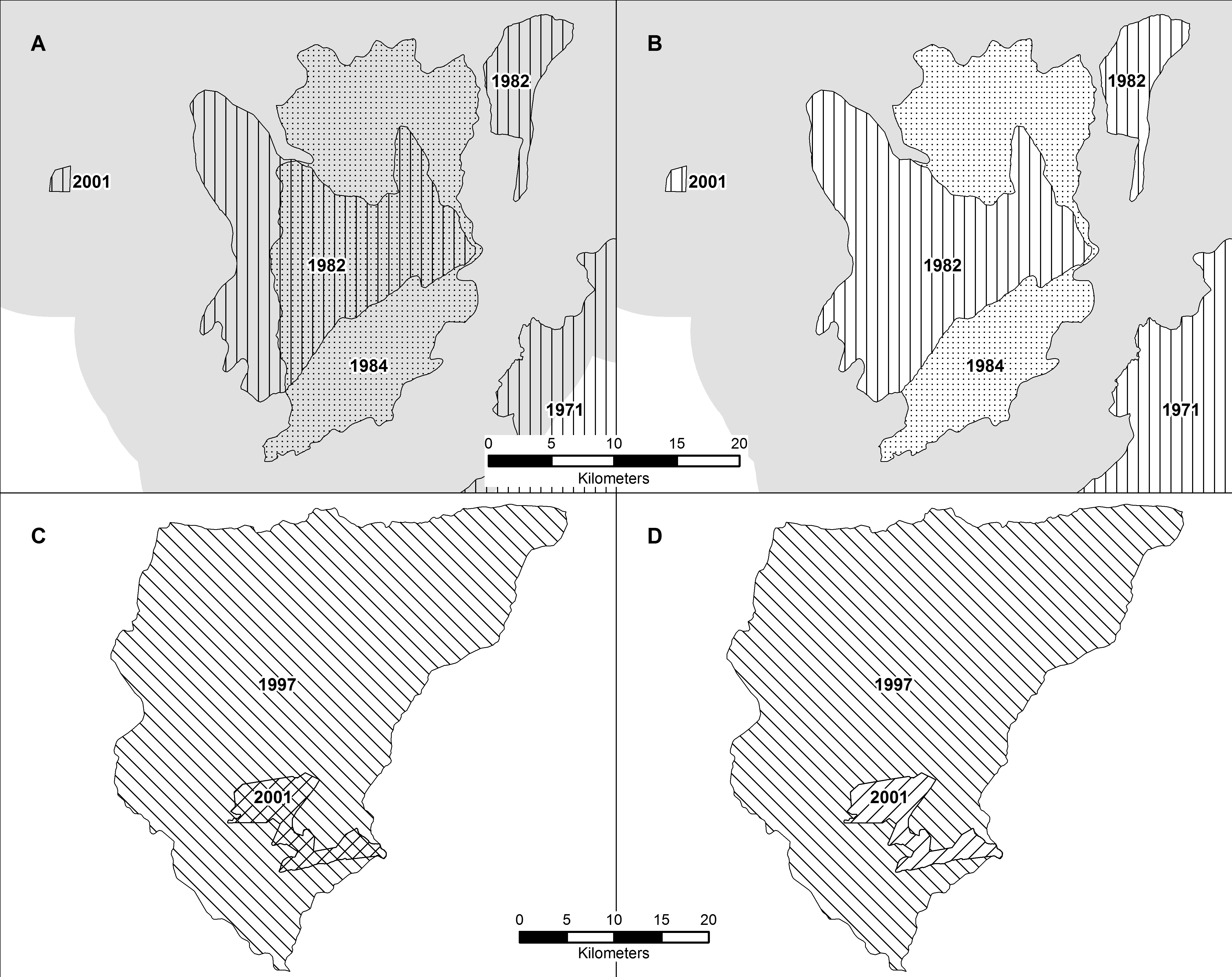

Supplement: Figure S1 — Examples of GIS treatment of spatial overlaps that are often found between forest reserves in Brazil: (A) a state-level reserve created in 1984 (dotted area) partially overlaps a federal reserve created in 1982 (hatched area), whereas the reserve buffer zone (shaded area) overlaps another reserve; (B) overlaps are manually removed, whereby the boundaries of the older reserve prevails; (C) a strictly protected reserve was created in 2001, partially overlapping a sustainable use reserve created in 1997; and (D) the overlap was then removed to maintain the more recent (and more restrictive) reserve in the sample. [file peerj-04-2206-s001.png]

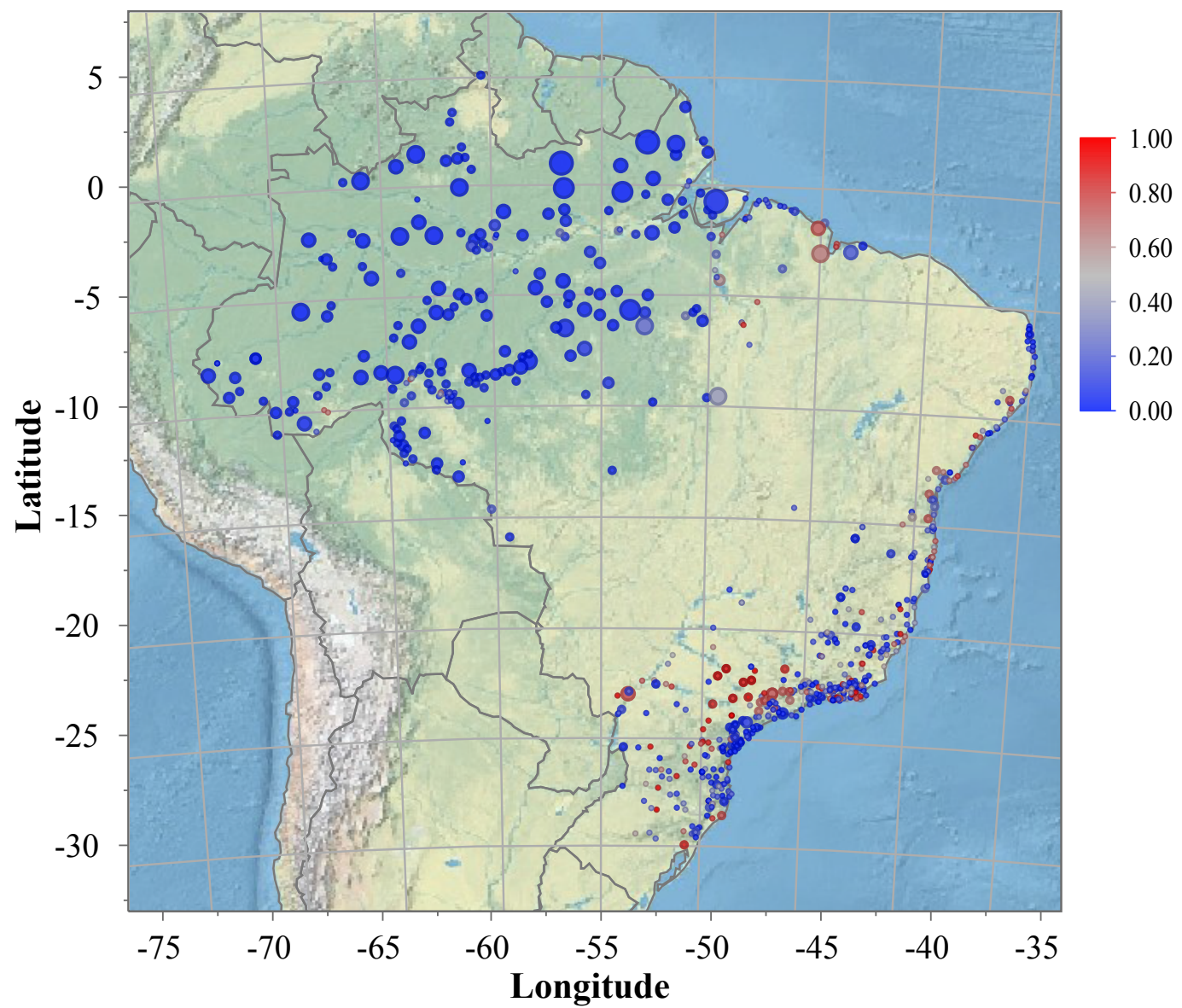

Supplement: Figure S2 — Distribution of all 788 reserves examined in this study across the Brazilian Amazon and the Brazilian Atlantic Forest biomes. Circle sizes are proportional to the log-transformed area (log10 x, hectares) of each reserve. Color gradient of reserve centroids indicate the degree to which their natural forest cover had been converted into other land uses (from blue to red indicating least to most degraded). [file peerj-04-2206-s002.pdf]

Cumulative area protected (ha)

$7 \times 10^7$   
 $6 \times 10^7$   
 $5 \times 10^7$   
 $4 \times 10^7$   
 $3 \times 10^7$   
 $2 \times 10^7$   
 $10^7$   
0

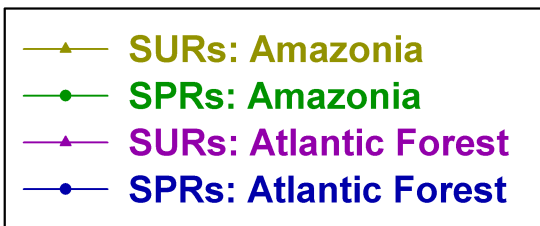

1910 1920

1960

1980

2000

Year of reserve decree

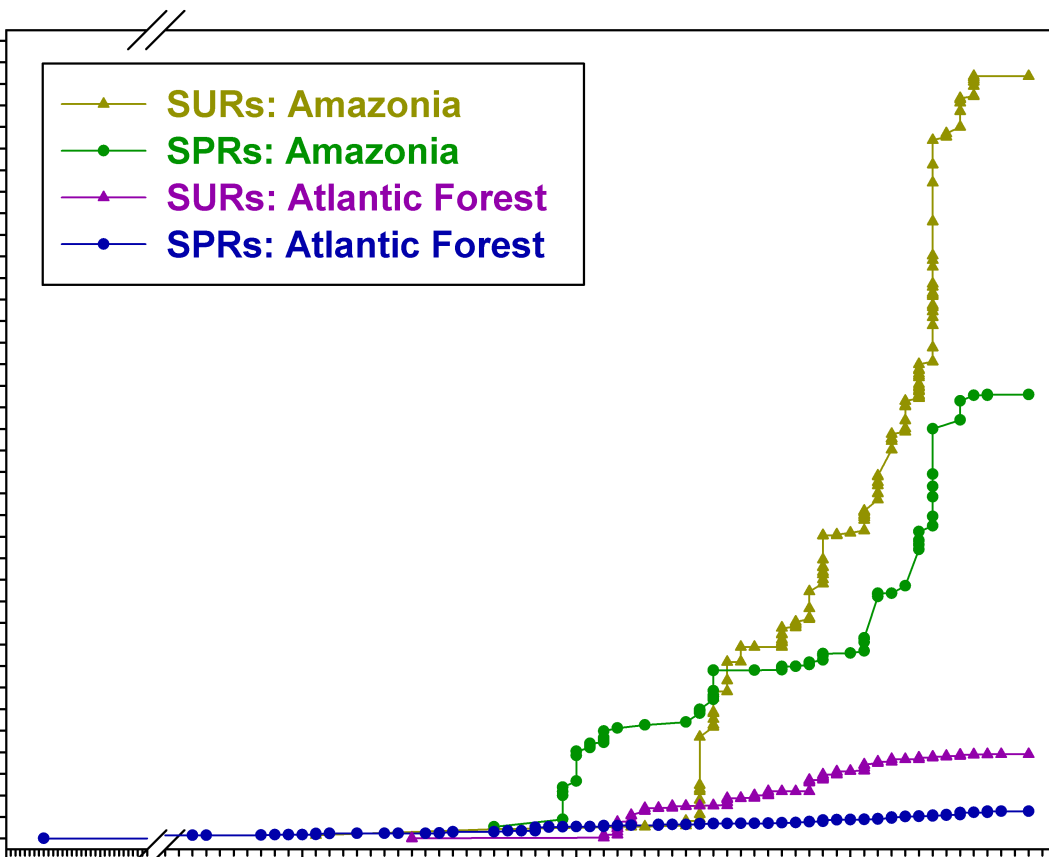

Supplement: Figure S3 — Temporal growth in total reserve acreage in Amazonia and the Atlantic Forest biomes, broken down by major reserve types (SUR, sustainable use reserves; SPR, strictly protected reserves) (data sourced from the CNUC database). [file peerj-04-2206-s003.pdf]

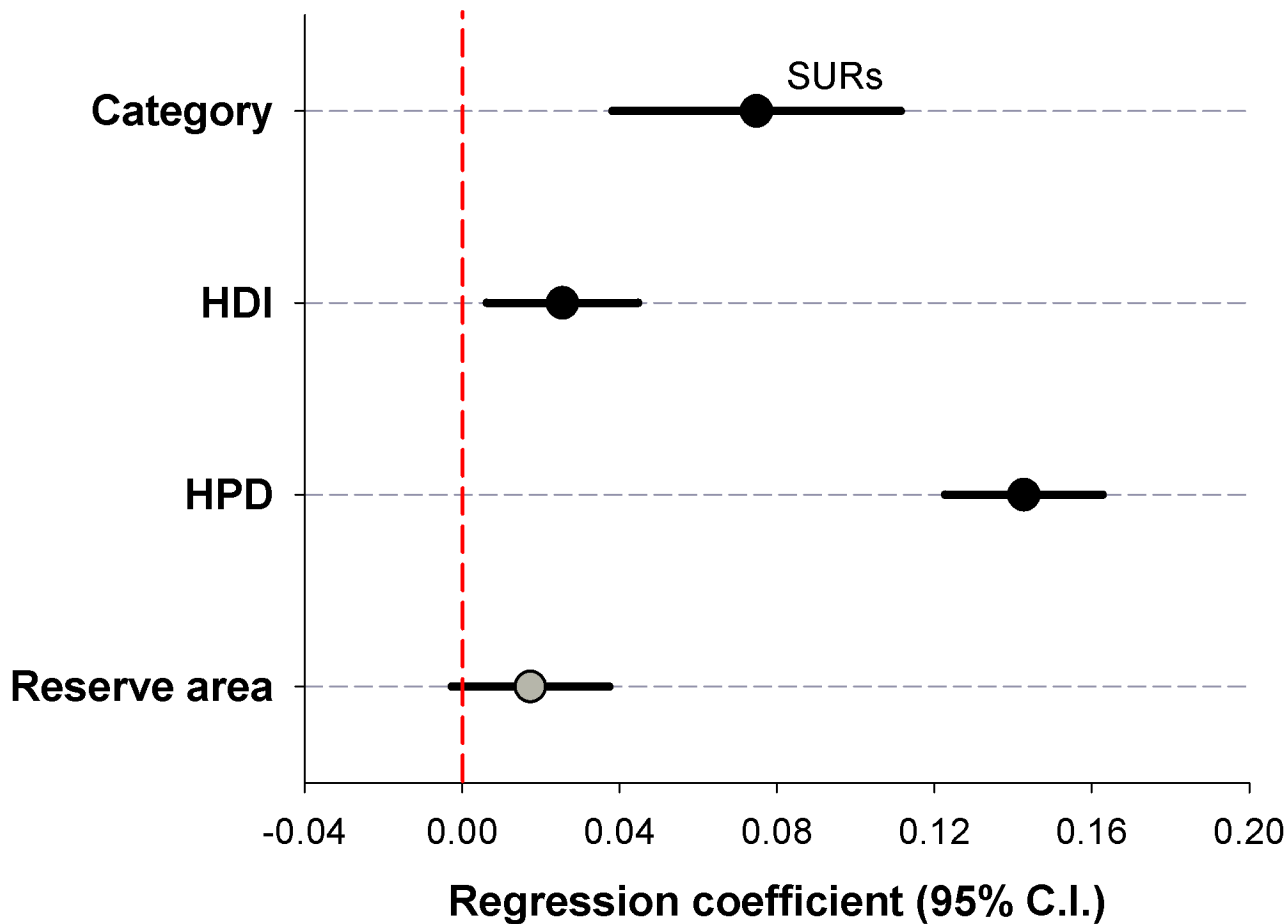

Supplement: Figure S4 — Coefficient estimates (±95% confidence intervals) showing the magnitude and direction of effect sizes of different reserve size and socioeconomic predictors of the degree to which 788 forest reserves had been converted to other land uses across the Brazilian Amazon and the Brazilian Atlantic Forest. Only variable retained in the most parsimonious final models are shown, including HPD (Human Population Density), HDI (Human Development Index) and major class of reserve category (SURs and SPRs). For a description of predictor variables, see Methods. [file peerj-04-2206-s004.pdf]
